# Mental disorders and heart diseases: from William Harvey to today

**DOI:** 10.1590/1516-3180.2017.1354110717

**Published:** 2017-05-29

**Authors:** Paulo Andrade Lotufo

**Affiliations:** I Medical doctor, Doctorate in Public Health and Full Professor, Department of Internal Medicine, Faculdade de Medicina da Universidade de São Paulo (FMUSP), São Paulo (SP), Brazil.

Coronary heart disease (CHD) is the leading cause of death worldwide.^1^ Despite the decline in CHD mortality rates for all social strata in Brazil, the burden of disease remains higher among the poor than among the rich.^2^ Nonetheless, since the 1950s, studies on cardiovascular epidemiology have successfully created a new agenda for this disease, which had until then been regarded as part of the fate of human existence or as an inescapable cause of death. In contrast to commonly held beliefs, these population-based studies showed that myocardial infarction and sudden cardiac death were the consequence of a myriad of identifiable causes.^3^ Since then, new reasoning to explain disease frequencies that differ from those of communicable diseases (i.e. one disease linked to one pathogen) has emerged. This “myriad of causes” received the name “risk factors”, which was the title of one of the first published papers communicating results from the Framingham Heart Study.^4^

The three major CHD risk factors of high cholesterol, hypertension and smoking habit have been well described since the 1960s.^5^ However, it has taken decades to implement effective medical treatment for reduction of high cholesterol and hypertension on a wide scale, and it is still insufficient for less affluent people.^6^ Despite warnings from the United States Surgeon General as early as 1963 and several actions coordinated by the World Health Organization, reduction of the smoking habit became a reality in the Western world only in the first decade of the 21^st^ century. The reason for this was the strong resistance from the tobacco industry.^7^

One different approach towards explaining the rising incidence rates of CHD has come from an old idea linking the brain and the heart. When William Harvey described the circulatory system for the first time, during the 17^th^ century, he warned that disorders of the brain or mental disturbances could impair the heart and the circulatory system.^8^ Stress within individuals’ daily lives has been correlated with heart diseases since the 1950s, but epidemiological tools to describe this are almost nonexistent.^9^

Two cardiologists in the United States launched the theory that one particular personality, the “type A behavioral pattern” was the cornerstone to understanding the CHD epidemic. Type A consisted of individuals who were highly competitive, ambitious, work-driven, time-conscious and aggressive. This theory was promptly accepted by the media after a famous book wrote by its proponents.^10^ However, only two prospective studies corroborated this hypothesis; the results were negative in all other studies.^11^

The fall of the type A behavioral pattern theory was due not only to scientifically-based risk factors. The not-so-happy end of this hypothesis was the revelation of yet another instance of interference from the tobacco industry in academic fields. Since people with Type A were smokers more often than were other individuals, the Philip Morris company funded several types of research addressing the type A behavioral pattern. The aim was to show that the smoking habit was only an “intermediary factor”, which was inserted in a pathway that had hypothetically begun with an unhealthy behavioral risk factor called type A and would end with myocardial infarction or sudden cardiac death.^12^

The type A behavioral pattern theory indeed helped the tobacco industry to avoid restrictions on cigarette advertising and sales. It also had a side effect: since this hypothesis linked CHD incidence to the behavior of rich white men, healthcare policy-makers worldwide did not consider CHD to be a public health priority for a long time, because of the false inference that heart diseases are not frequent among less affluent people.

Researchers working on the Brazilian Longitudinal Study of Adult Health (ELSA-Brasil) did not accept the type A behavioral pattern theory. Instead, they hypothesized that mental diseases are linked to heart disease, on the basis of several prospective studies addressing anxiety, depression and anger and their association with CHD.^13^ The baseline evaluation of ELSA-Brasil revealed that anxiety was related to prevalent cardiovascular diseases,^14^ to subclinical atherosclerosis (as measured from the intimal-media thickness of the common carotid artery)^15^ and to coronary calcification.^16^ Moreover, prescription of medicines for mental disorders was associated with low heart rate variability, which is a marker of adverse health outcomes.^17^ The frequency of depression was correlated with presence of diabetes.^18^

In conclusion, mental health disorders should be a permanent variable to be added to all epidemiological studies addressing cardiovascular diseases.
